# A preliminary investigation to explore the cognitive resources of physicians experiencing difficulty in training

**DOI:** 10.1186/s12909-017-0918-z

**Published:** 2017-05-15

**Authors:** Fiona Patterson, Fran Cousans, Iain Coyne, Jo Jones, Sheona Macleod, Lara Zibarras

**Affiliations:** 1 Work Psychology Group, Derby, UK; 2 Department of Neuroscience, Psychology and Behavoiur, University of Leicester, Leicester, UK; 3 School of Business and Economics, Loughborough University, Loughborough, UK; 4 Health Education East Midlands, Nottingham, UK; 5 City, University of London, London, UK

**Keywords:** Cognitive resources, International medical graduates, Physicians, Verbal comprehension, Working memory, Perceptual reasoning, PSU

## Abstract

**Background:**

Treating patients is complex, and research shows that there are differences in cognitive resources between physicians who experience difficulties, and those who do not. It is possible that differences in some cognitive resources could explain the difficulties faced by some physicians. In this study, we explore differences in cognitive resources between different groups of physicians (that is, between native (UK) physicians and International Medical Graduates (IMG); those who continue with training versus those who were subsequently removed from the training programme); and also between physicians experiencing difficulties compared with the general population.

**Methods:**

A secondary evaluation was conducted on an anonymised dataset provided by the East Midlands Professional Support Unit (PSU). One hundred and twenty one postgraduate trainee physicians took part in an Educational Psychology assessment through PSU. Referrals to the PSU were mainly on the basis of problems with exam progression and difficulties in communication skills, organisation and confidence. Cognitive resources were assessed using the Wechsler Adult Intelligence Scale (WAIS-IV). Physicians were categorised into three PSU outcomes: ‘Continued in training’, ‘Removed from training’ and ‘Active’ (currently accessing the PSU).

**Results:**

Using a one-sample Z test, we compared the referred physician sample to a UK general population sample on the WAIS-IV and found the referred sample significantly higher in Verbal Comprehension (VCI; z = 8.78) and significantly lower in Working Memory (WMI; z = −4.59). In addition, the native sample were significantly higher in Verbal Comprehension than the UK general population sample (VCI; native physicians: z = 9.95, *p* < .001, d = 1.25), whilst there was a lesser effect for the difference between the IMG sample and the UK general population (z = 2.13, *p *= .03, d = 0.29). Findings also showed a significant difference in VCI scores between those physicians who were ‘Removed from training’ and those who ‘Continued in training’.

**Conclusions:**

Our results suggest it is important to understand the cognitive resources of physicians to provide a more focussed explanation of those who experience difficulties in training. This will help to implement more targeted interventions to help physicians develop compensatory strategies.

## Background

During medical education, training and clinical practice, some physicians experience difficulty [1]. It is important to understand potential causes of difficulty, implement interventions, and support physicians through this process [2], since if they are not identified and supported, there may be negative consequences for their practice, patient care and safety. Across a range of medical specialties, physicians’ competence, and thus quality of patient care, is related to cognitive resources [3–6]. In essence the brain has finite processing power for various cognitive resources such as memory, attention and recognition and therefore as tasks become harder, performance can degrade [7]. In medicine, substantial cognitive resources are needed to learn the knowledge required to complete medical training and then apply this knowledge into clinical practice [8]. Similarly, once training has been completed, the rapid pace of development in medicine means that qualified doctors must constantly update their knowledge.

Previous research shows there are differences in cognitive resources between physicians who experience difficulties in training and clinical practice, and those who do not [9–11]. For example, Perry [12] found that physicians referred for remedial action scored significantly lower than a comparison group on a wide variety of cognitive ability tests; including picture arrangement, numerical attention and complex figure learning. Similarly, Yao and Wright [13] found that the three most common physician deficiencies related to cognitive resources (e.g. poor clinical judgement, insufficient medical knowledge, and inefficient use of time), leading to significant gaps in basic clinical knowledge, diagnoses and management of patients. There have been several different explanatory frameworks put forward to explain difficulties faced by both physicians [2, 14, 15] and medical students [16–19], and these have included not only cognitive resources, but also factors such as personal circumstance, learning style, personality and experience. Existing research suggests that differences in cognitive resources may provide an explanation for some of the difficulties faced by physicians. Some frameworks attempt to classify cognitive resource difficulties, with an aim to understand causality and guide trainers in effectively supporting physicians [20–22]. However, researchers have not yet converged on a set of cognitive resources that relate specifically to experiencing difficulty in medical training or clinical practice.

The Wechsler Adult Intelligence Scale (WAIS-IV) is one way of understanding a person’s cognitive resources. Intelligence as originally defined by Wechsler [23] is the “capacity to act purposefully, to think rationally, and to deal effectively with his [or her] environment”. The WAIS was designed based on intelligence theory, cognitive development and neuroscience and measures four key cognitive resources including verbal comprehension, perceptual reasoning, working memory and processing speed. Verbal Comprehension represents the ability to express thoughts verbally; to understand, analyse and interpret written information [23]; Perceptual Reasoning includes nonverbal reasoning, spatial processing and visual perception; Working Memory represents the ability to hold several pieces of information in the mind at once, and to manipulate and use this information [24], and Processing Speed relates to visual perception, scanning and hand-eye co-ordination [25].

In practice, treating patients uses many cognitive resources: accurate and efficient information processing is required with sustained attention, rapid decision making, and skilled execution of relevant actions [8]. Physicians must do this whilst facing competing priorities, managing distractions and interruptions; adding to already challenging tasks [26]. It is plausible therefore that physicians in difficulty may differ on some of these important cognitive resources; for example, the demand placed on a physician’s working memory (WM) is likely to be high; where WM provides temporary storage and manipulation of several pieces of information at once and so WM is needed for dealing competently with complex tasks [24]. A further consideration may be the additional cognitive resource demands required for physicians practicing in a second language [24]. Large-scale, meta-analytic studies have found differences in training success between native and International Medical Graduate (IMG) physicians (as measured by postgraduate examinations), with IMGs performing less well in comparison to native graduates [27–29]. As yet, there is no theoretical explanation for this phenomenon [28, 30] however, in developed countries there is continued dependence on IMGs to meet workforce needs [31, 32]. Therefore, it is important for countries internationally to better understand barriers that IMGs may face in completing medical training and practice; for example, the extent to which this is due to differences in specific abilities related to IMGs working in a second language.

Indeed, research relating to cognitive resource allocation theories [33] would imply that IMGs practicing in a second language are likely to have significantly increased working memory (WM) demands; for example, needing to correctly translate when communicating with others. This effect may become more pronounced when working under stress and in environments with high demands for cognitive resources, as in a medical context [34]. High demands are placed on the WM of medical physicians practicing in their own language [35–39], and those practicing in a second language may have the added burden of using WM capacity for translation processes. This in turn reduces the available WM capacity allocated to medical tasks and problem solving. Arguably, there may be other explanations for poorer clinical performance of IMGs related to the passivity in learning style in some non-Western cultures (rather than the critical analysis and challenge-based approach to learning strategies in Western learning strategies, [40, 41]). This may make the problem-solving nature of clinical practice more challenging for IMGs practising in a foreign country than for native-trained doctors.

In summary, physicians’ cognitive resources may significantly influence their performance in clinical practice. This study provides a preliminary examination of the cognitive resources of a sample of physicians referred for additional support due to experiencing difficulties. We address three key research questions:

1. *Are there differences in cognitive resources between physicians experiencing difficulties compared with the general population?*
2. *Are there differences in cognitive resources between native (UK) physicians and IMGs?*
3. *Are there differences in cognitive resources between physicians who continued with training and those who were subsequently removed from the training programme?*

## Method

### Setting

A secondary evaluation was conducted on an anonymised dataset provided by the East Midlands Training Support Service (TSS, as it was originally known at the time of the study) in the UK. TSS was a service provided by Health Education East Midlands for physicians having trouble with performance and progression in training. The dataset spanned from 2005 (when the TSS was established) to the end of May 2013. The TSS is now called the Professional Support Unit (PSU) so we will use this terminology throughout the rest of the paper. The PSU informs all users of the service that information about progression in training is gathered and that their data may be used for evaluation purposes. All data are stored in accordance with the Data Protection Act and were anonymised to remove identifying information.

### Participants and procedure

Data was available for 138 physicians who took part in an Educational Psychology assessment through PSU. Cognitive resources were assessed using the Wechsler Adult Intelligence Scale (WAIS). However, both WAIS-III and WAIS-IV were used during the period and given the changes between WAIS-III and WAIS-IV in calculating cognitive ability Index scores [25] we used only data available from the WAIS-IV. Therefore 15 people were omitted from the dataset. This resulted in a sample of 123 participants, spanning the different years of postgraduate training, with the most common grade at the time of referral being specialty training year three (*n* = 44), followed by specialty training year two (*n* = 23), specialty training year four (*n* = 10) and core training year two (*n* = 10), the rest of the sample (*n *= 36) included participants from foundation training, core training and specialty training. The sample included a mix of those who completed their medical qualification in the UK (*N* = 63; 33 males, 30 females), and those who were IMGs (*N* = 60; 39 males, 20 females); data was missing from one participant. For the IMG sample, countries where qualifications were obtained included India, Pakistan, Poland and Nigeria where English is likely to have been a second language. However, two participants were removed from the IMG sample since their places of qualification were New Zealand and Australia (meaning that English was unlikely to be a second language) resulting in *N* = 58 participants; 37 males, 20 females, and one participant for which this data was missing. The final sample was 121 participants, comprising 70 males and 50 females (data was missing from *n* = 1), with a mean age of 33.9 years (S.D. = 5.82 years). Referrals to the PSU were mainly based on problems with exam progression, difficulties in communication skills, organisation and confidence.

To compare our physician sample with a general population sample, we used the WAIS-IV UK validation sample, details of which can be obtained in the WAIS-IV manual [25]. This sample included 270 people (52.5% were female and 47.5% were male), their mean age was 44.33 years (S.D. = 19.14 years). The WAIS-IV manual highlights that this sample closely matches the adult population relating to distribution of demographic variables in terms of age, gender, ethnicity [25].

To compare cognitive resource scores across successful/unsuccessful training groups, physicians were categorised into groups of three possible PSU outcomes. ‘Continued in training’ (*n* = 57) represents those physicians who achieved Certificate of Completion of Training (CCT) and/or remained in the programme after receiving remedial support. ‘Removed from training’ (*n* = 15) represents those physicians who had their National Training Number removed, voluntarily left the programme, or were suspended by the UK’s regulator (General Medical Council). Finally, ‘Active’ (*n* = 49) represents physicians currently in the system and accessing support through the PSU at the time the study was conducted.

### Measures

Physicians completed the WAIS-IV, which is designed to provide a detailed assessment of the cognitive resource of adolescents and adults aged between 16 to 90 years, and is used regularly in organisational and clinical settings. The WAIS assesses specific cognitive resources, and comprises four index scores: *Verbal Comprehension Index* (VCI; 92 items), *Perceptual Reasoning Index* (PRI; 117 items), *Working Memory Index* (WMI; 76 items) and *Processing Speed Index* (PSI; 68 items); it takes approximately 75 min to complete. Evidence of the reliability and validity of the WAIS-IV has been reported [42]. Table 1 outlines how the four WAIS-IV Index scores relate to the physicians’ job role.

**Table 1:** WAIS-IV index scores: capacities assessed and relevance to physicians’ job role^a^

WAIS Index Primary Capacities Targeted for Assessment	Relevance to Physicians’ Job Role
Verbal Comprehension Index	Verbal reasoning and conceptualising ability – synthesising and modelling ideas. Lexical knowledge – receptive and expressive vocabulary. General societal knowledge.
Retrieval of verbal information from long-term storage	
Reasoning with verbal information	*For example: being able to explain information clearly to patients and colleagues; being able to remember the diagnostic criteria for different medical conditions; orally presenting cases and justifying decisions made in relation to care.*
Perceptual Reasoning Index	Interpreting and reasoning with pictorial, diagrammatic, schematic and graphical information – problems of omission and commission in pattern recognition and matching. Integration of two and three dimensional data. Hypothetico-deductive reasoning.
Reasoning with nonverbal visual stimuli	
	*For example: being able to interpret nonverbal information on a patient,* e.g. *an x-ray or scan; being able to interpret and use presenting visual symptoms to contribute towards making an informed diagnosis.*
Working Memory Index	Short-term auditory sequential memory for holding complex patient and colleague information in mind. Central executive working memory for mental computation, whilst drawing on information in long term memory, and then executing conclusion in ordered and logical manner. Personal organisation.
Initial registration and holding of information (sometimes referred to as short term memory)	
The mental manipulation of information that is being held in mind (often referred to as working memory)	*For example: being able to listen to a patient tell you their symptoms, hold this information in mind, whilst combining it with other information from alternative sources,* e.g. *past medical history, information from colleagues or family members, whilst also bringing to mind the diagnostic criteria for different possible conditions.*
Processing Speed Index	Proofing and clerical checking. Working against the clock in time pressured situations without error. Transcribing. Fine motor precision and accuracy. Visual scanning and tracking. Holding data in the visual-spatial sketchpad aspect of working memory. Dealing with cognitive noise (Stroop).
Processing speed with nonverbal, visual stimuli	
	*For example: being able to quickly scan information about a patient to identify relevant details; proofing reports or clinical notes; analysing nonverbal information (*e.g. *figures, pictures, images) during a time-pressured exam.*

^a^Adapted from table created by Michael Lock Consultant Psychologists, 2013 and information from ‘Primary Capacities Targeted for Assessment’ taken from Lichtenberger and Kaufman (2013, Appendix B1)

## Results

To explore the first research question, *are there differences in cognitive resources between physicians experiencing difficulties compared with the general population?* we examined the means and SDs for the WAIS-IV Index scores and compared this to data taken from the UK general population sample of 270 individuals (see Table 2 for means and SDs for WAIS-IV Index Scores for physician and UK general population samples).

**Table 2:** Means, SDs and one-sample Z statistics for the current physician sample and the UK WAIS-IV general population sample

	UK General Population Sample			Current sample (IMG & UK Combined)				UK Physician Sample				IMG Physician Sample			
	N	Mean	SD	N	Mean	SD	z	N	Mean	SD	z	N	Mean	SD	z
VCI	270	100.10	14.18	116	111.66	14.16	8.78	63	117.89	12.66	9.95	53	104.26	12.23	2.13
PRI	270	103.82	14.45	113	103.91	14.12	0.06	60	108.43	13.56	2.47	53	98.79	13.06	−2.53
WMI	270	104.17	15.97	119	97.45	9.85	−4.59	62	98.03	9.51	−3.03	57	96.82	10.24	−3.47
PSI	270	102.51	14.63	114	101.07	14.85	−1.05	59	102.51	16.38	−.01	55	99.53	12.97	−1.52

*VCI* verbal comprehension index, *PRI* perceptual reasoning index, *WMI* working memory index, *PSI* processing speed index

The WAIS-IV index scores are standardised, with a mean of 100 and standard deviation of 15. This is the standard format for tests of intelligence such as the WAIS-IV [23, 25] and is generally conducted in this way so that scores can be more readily compared to those from other tests and to aid interpretation by lay-users. The UK general population (WAIS-IV UK norm group) sample parameters are known (accessible from the manual [25], and outlined in Table 2). A one sample z-test was used to compare the physician sample to the UK general population sample on the WAIS-IV. Given an alpha level of.05 and using a two-tailed design, if z is +/−1.96, the referred physician sample is significantly different from the UK general population sample. We also explored Cohen’s *d* statistic to examine the size of the difference, where as a rule of thumb, 0.2 is a small effect size; 0.5 is medium; 0.8 is large and 1.2 is a very large effect [43].

Results in Table 2 show that the physician sample does not differ significantly from the UK general population sample in Perceptual Reasoning (PRI) or Processing Speed (PSI). However, the referred sample is significantly higher in Verbal Comprehension (VCI; z = 8.78, *p* < .001, *d* = 1.25) and significantly lower in Working Memory (WMI; z = −4.59, *p* = .01, *d* = .29), than the UK general population sample.

To explore the second research question *are there differences in cognitive resources between native (UK) physicians and IMGs?* we split the sample by place of medical qualification (native versus IMG). Findings show that the native physician sample is significantly higher in Verbal Comprehension than the UK general population sample (with a very large effect). The IMG sample is also significantly higher than the UK general population sample, but to a lesser degree, showing a small effect size (VCI; native physicians: z = 9.95 *p* < .001, *d* = 1.25; IMGs: z = 2.13 *p* = .03, *d* = .29). This implies that any difference between VCI scores in the UK general population sample and the physician sample are explained to a greater degree by the native physicians.

In addition, the native physician sample is significantly higher on Perceptual Reasoning (PRI; z = 2.47, *p* = .01, *d* = .32), whereas the IMG physician sample is significantly lower (z = −2.53, *p* = .01, *d* = −.35) than the UK general population sample. Both the native and IMG physician samples were significantly lower on Working Memory than the UK general population sample (WMI; native: z = −3.03, *p* = .002, *d* = −.38; IMG: z = −3.47, *p* < .001, *d* = −.46). Neither the native nor the IMG physicians had significantly different Processing Speed (PSI) scores to the UK general population sample.

Finally, to explore the third research question *are there differences in cognitive resources between physicians who continued with training and those who were subsequently removed from the training programme?* a 3 × 2 multivariate analysis of variance (MANOVA) was used to test the differences in WAIS-IV Index scores between place of qualification and PSU outcome. The equality of covariance matrices using Box’s test indicated the assumption of homogeneity was met (M = 18.48, *p* = .65). Table 3 illustrates the descriptive statistics by training outcome and place of training for each of the WAIS-IV scores.

**Table 3:** Means and SDs for WAIS-IV index scores for PSU outcome and place of qualification

	VCI				PRI				WMI				PSI			
	UK		IMG		UK		IMG		UK		IMG		UK		IMG	
	Mean	SD	Mean	SD	Mean	SD	Mean	SD	Mean	SD	Mean	SD	Mean	SD	Mean	SD
Continued in training	117.25	10.60	106.53	11.68	108.44	12.97	102.88	14.40	100.59	8.71	97.29	8.64	100.69	17.62	98.82	11.88
Removed in training	107.00	8.16	100.20	11.25	103.00	13.40	97.80	13.67	97.60	8.44	99.80	11.71	103.40	13.80	97.00	10.61
Active	122.05	14.11	103.89	12.93	108.32	14.02	94.42	13.22	95.84	10.48	93.89	9.10	102.32	13.08	98.48	13.36

*VCI* verbal comprehension index, *PRI* perceptual reasoning index, *WMI* working memory index, *PSI* processing speed index

Results showed that there was no significant PSU outcome X place of qualification interaction where Pillai’s trace V = 0.53, F(8, 186) = 0.64, *p* = .74, partial ETA^2^ = .03. However, we explored each factor separately, the PSU outcome and the place of qualification, to identify whether the WAIS-IV scores were differentially influenced by these factors.

For PSU outcome, Pillai’s trace approached significance on WAIS-IV index scores, V = 0.16, F(8, 186) = 1.95, *p* = .06, partial ETA^2^ = .08, although for the same effect, Roy’s largest route indicated a significant effect Θ = 0.12, F(4, 93) = 2.92, *p* = .03. Separate univariate ANOVAs on index scores revealed a significant effect only on Verbal Comprehension (VCI); F(2, 113) = 4.42, *p* = .01, partial ETA^2^ = .07. Contrasts using the ‘continued in training’ group as the referent category highlighted that the significant differences in Verbal Comprehension scores are between those ‘removed from training’ and those who ‘continued in training’ (mean difference = 11.90, *p* = .004, 95% CIs 3.96 to 19.84).

When considering place of qualification, Pillai’s trace showed a significant effect on WAIS-IV index scores, V = 0.20, F(4, 92) = 5.80, *p* < .001, partial ETA^2^ = .20. Separate univariate ANOVAs revealed significant effects on Verbal Comprehension (VCI, F(1, 115) = 34.39, *p* < .001, partial ETA^2^ = .23) and on Perceptual Reasoning (PRI, F(1, 112) = 14.72, *p* < .001, partial ETA^2^ = .12). In both cases IMGs scored on average lower than those who trained in the UK.

## Discussion

This study is a first step towards understanding the cognitive resources of physicians referred for support due to difficulties experienced during training. Overall, our results indicate that the physicians in our sample had similar Perceptual Reasoning and Processing Speed to the general UK population sample; however, they were significantly higher in Verbal Comprehension and significantly lower in Working Memory. Considering the profile of the sample of referred physicians, they can be described as ‘Average’ in Perceptual Reasoning, Processing Speed and Working Memory, and ‘Higher than Average’ in Verbal Comprehension [23]. However, research elsewhere suggests that most physicians typically perform within the above average to superior range in these cognitive resources [12, 44]. Therefore, our findings may suggest that this sample of physicians is performing at a lower level of cognitive functioning than expected. IMG physicians were significantly lower in their Verbal Comprehension and Perceptual Reasoning than those trained in the UK. Furthermore, physicians who continued in training after receiving support scored significantly higher in Verbal Comprehension than those who were removed from training. Since there was no training outcome by place of qualification interaction, it is not the case that IMGs who were removed from training are significantly lower in Verbal Comprehension than other groups.

### Working memory ability

Findings showed that WM was the lowest of all index scores in the physician sample. This does not mean that the physicians have significant problems in WM, since they are performing within the average band. Rather, because WM represents the ability to hold several pieces of information in the mind at once, and to manipulate and use this information [24], lower scores may represent limitations in a physician’s clinical practice. Since a physician’s performance requires rapid processing of patient information and decision making, lower WM could mean that physicians may experience difficulties in identifying the correct decisions.

This latter notion could be explained with reference to dual-processing cognitive theories, which suggests two types of cognitive processes: one that is automatic and unconscious and the other is controlled and conscious. WM capacity is known to moderate the impact of automatic and controlled processes on self-regulatory behaviour [45]. Controlled processing is cognitively demanding and, in attention-demanding circumstances (such as during clinical practice), high WM capacity promotes the effortful and intentional behaviours of controlled processing, and also inhibits effortless, impulsive and potentially less desired behaviours seen in automatic processing. Experienced physicians generally formulate diagnostic decisions using automatic processing and only in novel/challenging situations would they access controlled processing [36]. Since the cognitive load for physicians is often high, and they operate in challenging situations, lower WM resources means less access to controlled processing and more likelihood that decisions will be based on automatic processing. This may lead to inappropriate or inaccurate decisions or behaviours. Further, the high cognitive demands of the physician’s role may be intensified for IMGs who are less familiar with the local health service. However, with appropriate training interventions WM could be improved, for example WM training has been conducted successfully in older adults where significant improvements in WM are seen even 8 months after the intervention [46]. Practically, this implies that earlier identification of struggling physicians would significantly benefit trainees and relevant educational interventions could support them appropriately.

### Verbal comprehension ability

Our findings showed that Verbal Comprehension (the ability to express thoughts verbally; to understand, analyse and interpret written information [23]) differentiates those who continued in, and those who were removed from, training; as well as native and IMG physicians. A physician lower in Verbal Comprehension may experience problems explaining information clearly; when verbally presenting cases; and when justifying decisions, especially under pressure. This may attenuate a physician’s clinical performance and progression (e.g. clinical skills, communication skills, exam performance and patient interaction). This is supported by the fact that most physicians were referred because of problems in postgraduate examination performance (which requires a high level of Verbal Comprehension), and difficulties in communication skills and organisation.

Lower scores for IMGs in Verbal Comprehension than native physicians may imply that performance issues are the result of language/accent difficulties. However, as there were no interaction effects, Verbal Comprehension in IMGs does not fully explain progression in training. Individuals whose first language is not English may initially experience problems when expressing thoughts and explaining information clearly [27] and may also over-estimate the language capabilities of others or medical understanding amongst patients (since many semi-technical terms are used in general discourse, such as “migraine” [47]); and inappropriately use overly-technical language.

Indeed, even IMGs whose first language is English may struggle to understand accent and colloquial language [48, 49]. In our sample, data were not available for participants’ native language so we could not substantiate this. Nevertheless, practising medicine in the UK requires an understanding of the nuances of British language (including humour and irony), which may differ across English-speaking nations. Consider for example the different terminology and phrasing used by Britons compared to Americans; therefore we may infer that some conversation may be challenging for IMGs, even if their first language is English.

### Perceptual reasoning ability

A possible explanation for the significant differences in PRI for the native physician sample (higher than the UK general population sample) and IMGs (lower than the UK general population sample) is the difference in learning methods between UK- and non-UK-trained doctors. Problem-Based Learning (PBL) is the prevalent approach to teaching in Western cultures [50], which enhances learning by focusing on problem solving in a self-directed and reflective way [51]. PBL positively impacts students’ abilities to apply science knowledge and transfer problem-solving skills to ‘real-world’ situations, across both medical and non-medical contexts [52], and has been found to prepare graduates with the knowledge and skills needed to practice in a complex healthcare system [53]. However, the PBL approach to learning is rooted in Western culture [40, 54], and other cultures tend to be focussed around teacher-led learning. Therefore, students from non-Western cultures may feel uncertain about the independence required for self-directed and problem-based learning [40] and these cultural differences in teaching and learning style may explain the observed differences between IMGs and UK-trained doctors in Perceptual Reasoning. The PRI includes problem solving, placing missing parts into an uncompleted picture, and the ability to differentiate between essential and non-essential details. These are abilities that PBL aims to promote, whereas in teacher-led learning, students learn what is taught to them by ‘experts’, rather than learning in a self-directed way [55].

Research by Frambach and colleagues [40] found that students across different cultures increasingly internalised the principle of self-directed learning as they progressed through their education and training, suggesting that Perceptual Reasoning skills may develop over time with more PBL-based approaches. Therefore, appropriately designed interventions may increase the Perceptual Reasoning ability of physicians experiencing difficulties, via increased PBL-style learning. Future research should investigate the suitability of PBL and other interventions for increasing Perceptual Reasoning ability in physicians experiencing difficulty.

### Implications

Our results are a first step towards understanding the cognitive resources of physicians experiencing difficulty and this may provide a more focussed explanation of difficulties in training, and help to implement targeted interventions for physicians to develop compensatory strategies. The recent literature on cognitive resources training (CT, also known as ‘brain training’) may provide some insights into possible interventions for physicians facing difficulties. CT is defined as “an intervention providing structured practice on tasks relevant to aspects of cognitive functioning” using standardized tasks and is “intended to address cognitive function and/or cognitive impairment directly” ([56] p.3). Although the majority of research in this area has focused on elderly participants, the medical context with healthy and comparatively young adults may be an area for exploration. CT approaches in a medical context may include: early testing for cognitive ability, rehabilitation for minor cognitive resource problems [57], development of critical thinking skills [58] and WM training [59]. Ultimately it is important to develop interventions for physicians because if left to struggle they may develop further problems which inhibit their progress as effective clinicians. Early interventions would prevent significant costs to the physician, patients and the health service as a whole.

### Limitations

As with any research, there were a number of limitations of the current study that should be noted. First, we were unable to compare WAIS-IV scores with those of a control group of non-referred physicians. However, the UK working population norm group was considered a good comparison group in this context. Second, we did not have data on why physicians were removed from training. This data was not available for data protection reasons. It could be that a subset of individuals within this group left the training programme due to reasons other than those relating to cognitive functioning (e.g. for social, personal or financial reasons). Future research could expand upon our preliminary findings to separate out the causes of physicians being removed from training. Finally, we did not have information regarding whether English was a first language for the participants in our sample. To further substantiate our findings, future research is needed to compare the cognitive resources and clinical performance of IMGs whose first language is English, and those for whom English is a second language. Nevertheless, we believe that this paper highlights some key areas to be considered for physicians in difficulty.

## Conclusions

This preliminary study extended empirical research relating to physicians experiencing difficulties in training. Our findings may go towards explaining some of the difficulties physicians faced. Our sample was lower than may be expected in WM, which could lead to limitations in physicians’ performance when making decisions in a fast-paced environment. Verbal Comprehension ability was significantly different between those who continued in, and those who were removed from, training; as well as between native and IMG physicians. Furthermore, while native graduates were significantly higher in Perceptual Reasoning than the UK general population sample, IMGs were significantly lower, which may be attributable to differences in learning styles across countries and cultures. Future research is necessary to expand upon these initial findings, to assess whether the cognitive resources in this study represent a potential taxonomy to explain difficulties faced by physicians in wider samples.
